# Supplementary material for: Transmembrane coupling of liquid-like protein condensates
Source: Nat Commun. 2023 Dec 4;14:8015. doi: 10.1038/s41467-023-43332-w (PMC10696066; doi:10.1038/s41467-023-43332-w)
Supplement: Supplementary file 1 — Supplementary Information [file 41467_2023_43332_MOESM1_ESM.pdf]

Supplementary Information

Transmembrane coupling of liquid-like protein condensates

Yohan Lee[1§], Sujin Park[2§], Feng Yuan[1], Carl C. Hayden[1], Liping Wang[3], Eileen M. Lafer[3], Siyoung Q. Choi[2] and Jeanne C. Stachowiak[1,4]*

[1]Department of Biomedical Engineering, The University of Texas at Austin, Austin, TX, USA.

[2]Department of Chemical and Biomolecular Engineering, Korea Advanced Institute of Science and Technology (KAIST), Daejeon, Republic of Korea.

[3]Department of Biochemistry and Structural Biology, The University of Texas Health Science Center at San Antonio, San Antonio, TX, USA.

[4]Department of Chemical Engineering, The University of Texas at Austin, Austin, TX, USA.

[§]These authors contributed equally.

*To whom correspondence should be addressed: jcstach@austin.utexas.edu

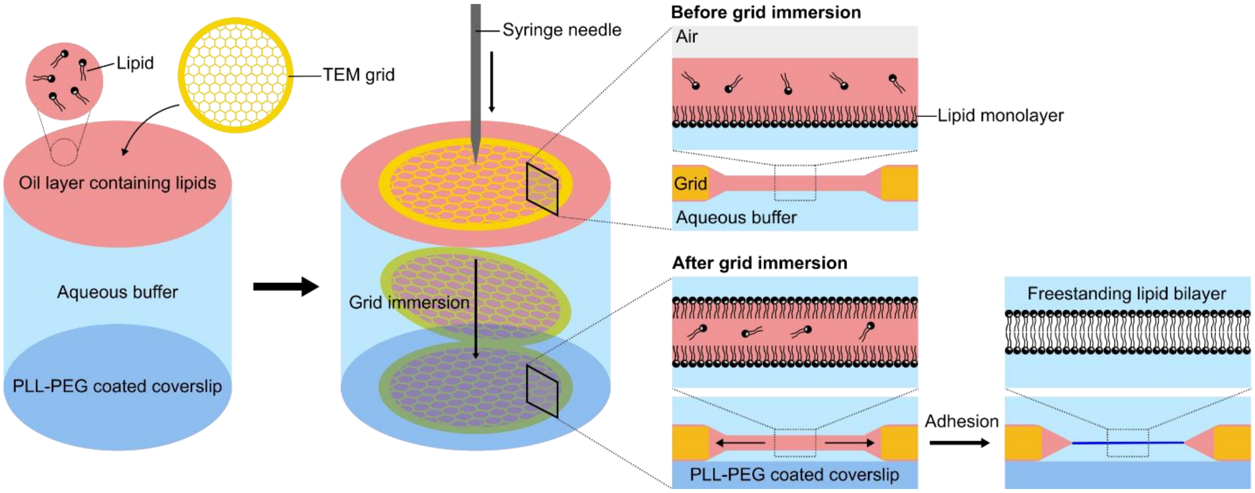

Supplementary Figure 1. Schematic of freestanding planar lipid bilayer formation. Initially, a TEM grid with hexagonal holes was placed onto an oil layer containing lipids, leading to the formation of a thin oil film within each grid hole with a lipid monolayer at the oil/buffer interface (before grid immersion). Next, the grid was immersed into the aqueous buffer using a syringe needle. Then, another lipid monolayer was formed at the oil/buffer interface. As the oil film in the grid hole became thinner, owing to oil draining back to the grid, spontaneous adhesion between the two lipid monolayers occurred, leading to a freestanding lipid bilayer (After grid immersion).

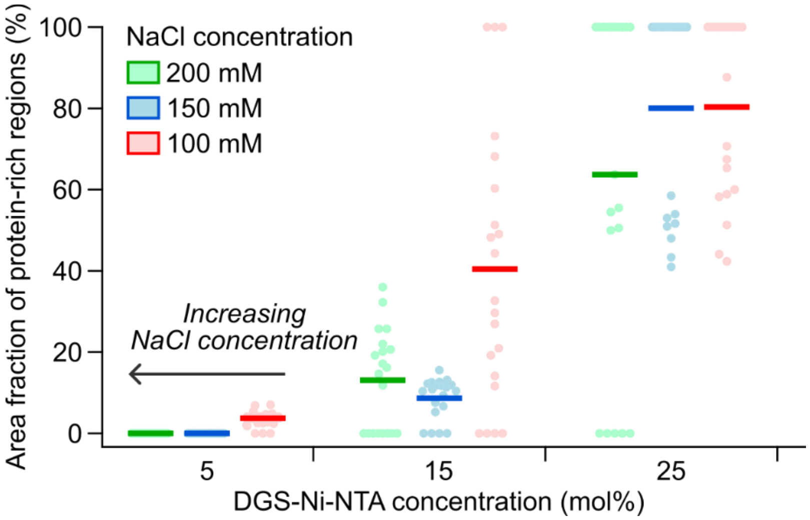

Supplementary Figure 2. Area fraction of protein-rich regions as a function of NaCl concentration and DGS-Ni-NTA concentration. A total of 20 lipid membranes were analyzed from three independent experiments for each condition. Bars represent the average area fraction for each case. Membrane composition: 75-95 mol% DOPC, 5-25 mol% DGS-Ni-NTA. 1 μM of his-RGG labeled with Atto 488 was used.

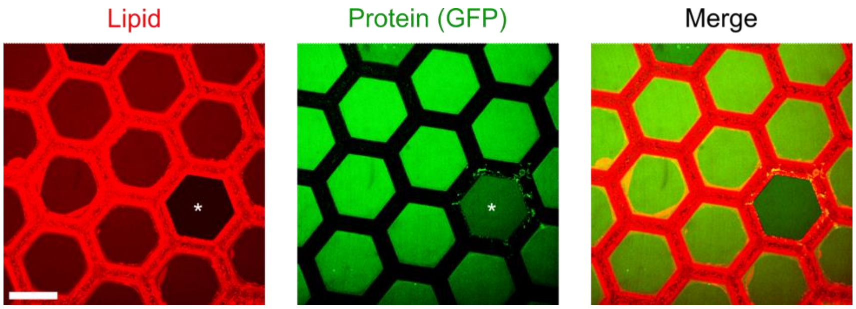

Supplementary Figure 3. Homogeneous binding of GFP to freestanding planar membranes. Representative microscopic images after adding 1 µM of his-GFP to the membrane. Note that the fluorescence intensity of a vacant hole (marked with an asterisk) is weaker than the surrounding membranes in the lipid and protein channels. Membrane composition: 85 mol% DOPC, 15 mol% DGS-Ni-NTA, and 0.5 mol% Texas Red-DHPE. Buffer: 25 mM HEPES, 200 mM NaCl, pH 7.4. Scale bar, 100 µm.

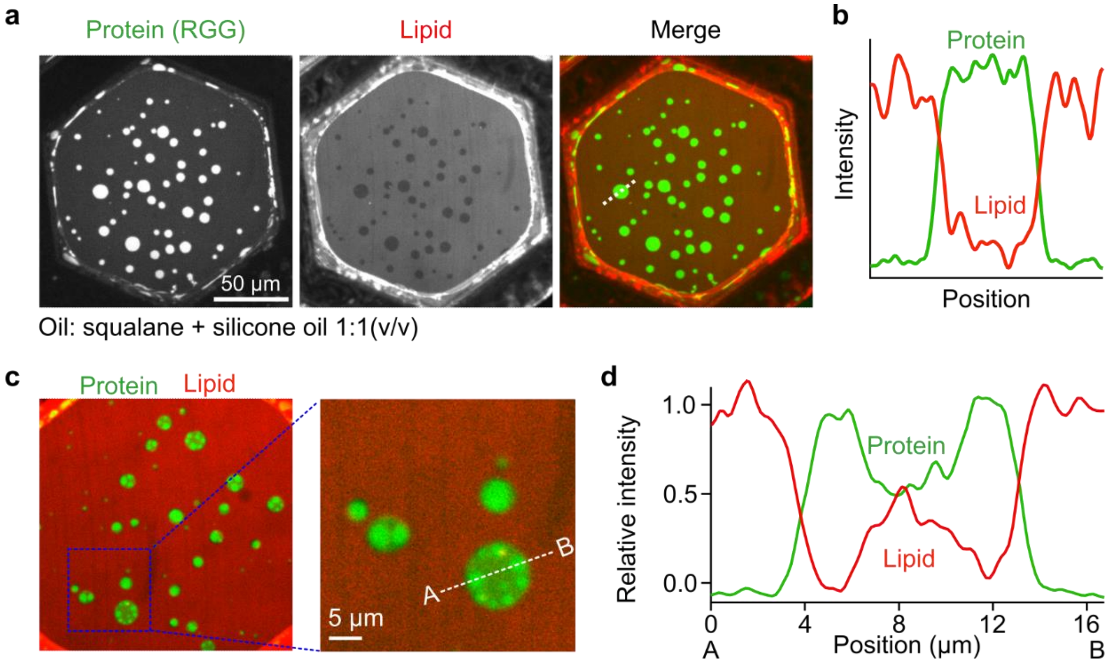

Supplementary Figure 4. Lipid probe partitioning and transbilayer coupling of RGG condensates on solvent-free lipid membranes. a, Representative images after adding 1 µM of his-RGG, labeled with Atto 488, to the membrane. The membrane could be considered as having an essentially solvent-free hydrophobic core since squalane ($C_{30}H_{62}$), a hydrocarbon with a bulky structure that does not easily enter the bilayers, was used instead of hexadecane ($C_{16}H_{34}$). Scale bar, 50 µm. **b,** Fluorescence intensity profile along the dotted white line in the merged channel in **a**, where green and red lines represent the intensity from the protein and lipid channels, respectively. **c,** Representative image showing transbilayer domain coupling. A region of interest within the blue dotted square is magnified. Scale bar, 5 µm. **d,** Intensity profiles along the dotted white lines in the magnified image in **c**. Membrane composition: 85 mol% DOPC, 15 mol% DGS-Ni-NTA, and 0.5 mol% Texas Red-DHPE. Buffer: 25 mM HEPES, 100 mM NaCl, pH 7.4.

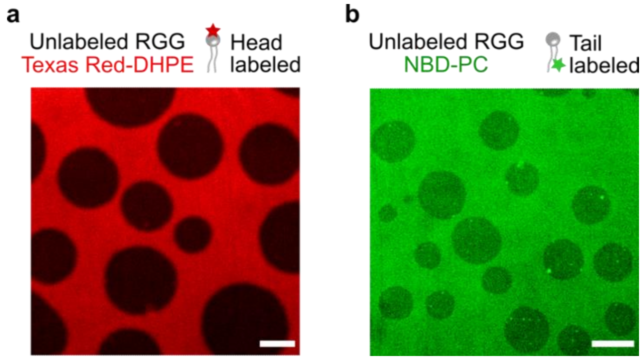

Supplementary Figure 5. Lipid probe partitioning also occurs when condensates consist of unlabeled RGG. Representative microscopic images in lipid probe channels, Texas Red-DHPE (**a**) and NBD-PC (**b**), after adding 1 µM of unlabeled his-RGG. Membrane composition: 85 mol% DOPC, 15 mol% DGS-Ni-NTA and 0.5 mol% lipid probe. Buffer: 25 mM HEPES, 50-100 mM NaCl, pH 7.4. Scale bars, 10 µm.

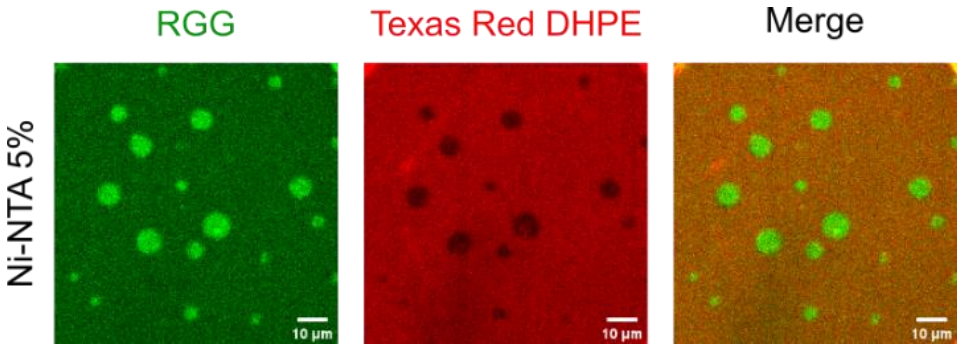

Supplementary Figure 6. Lipid probe exclusion from protein-rich regions with low DGS-Ni-NTA concentration in the membrane. Membrane composition: 95 mol% DOPC, 5 mol% DGS-Ni-NTA with 0.5 mol% Texas Red DHPE. Buffer: 25 mM HEPES, 100 mM NaCl, pH 7.4. 1 µM of his-RGG labeled with Atto 488 was used. Scale bar, 10 µm.

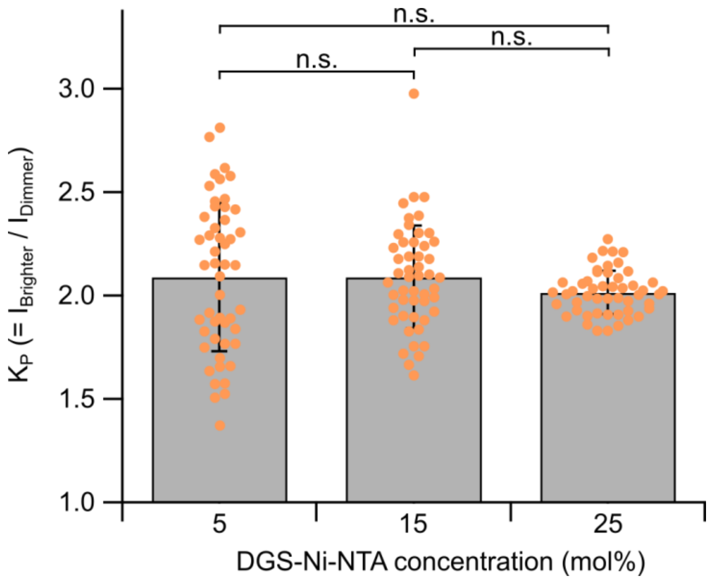

Supplementary Figure 7. Partition coefficients (K_P) of lipid probe as a function of DGS-Ni-NTA concentrations in the membrane. Data are presented as mean values ± SD (n = 50). Membrane composition: 75-95 mol% DOPC, 5-25 mol% DGS-Ni-NTA with 0.5 mol% Texas Red DHPE. 1 µM of unlabeled his-RGG was used. Brackets show statistically significant comparisons using an unpaired, two-tailed Student's t-test (n.s. indicates a difference that was not statistically significant).

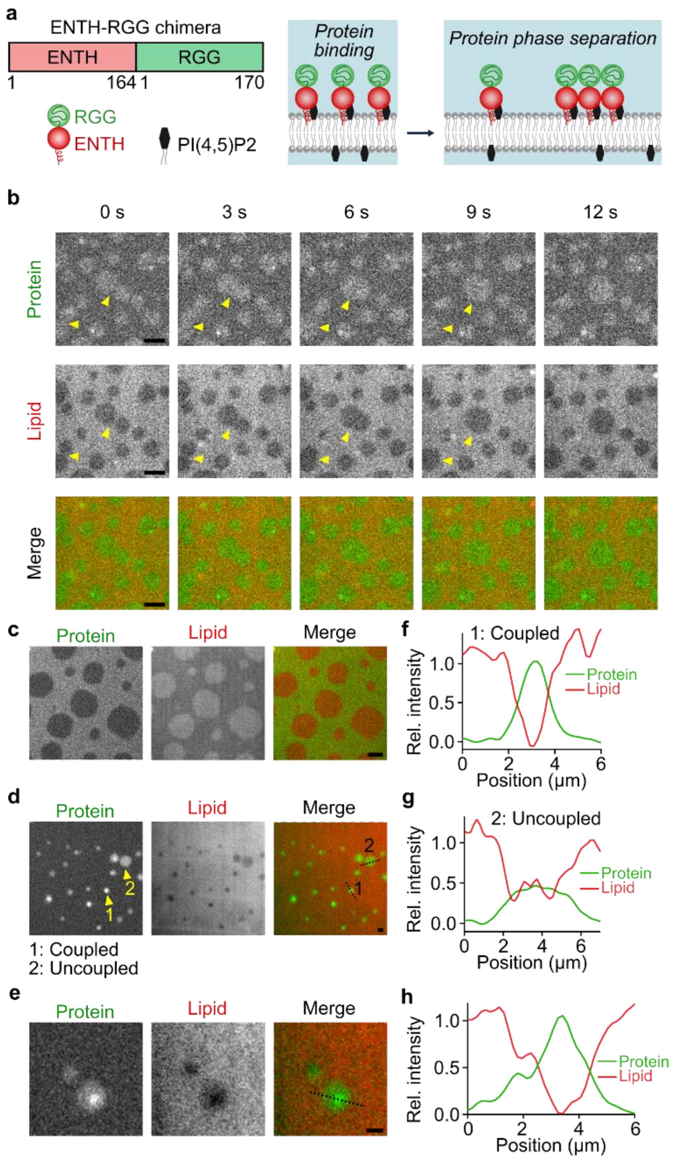

Supplementary Figure 8. Phase separation of ENTH-RGG protein chimera on the membrane. a, Schematic of recombinant protein chimera ENTH-RGG and its binding and phase separation on the membrane through interactions between ENTH domain and PI(4,5)P2 lipids. **b,** Images showing fusion of protein-rich regions. Yellow arrowheads indicate fusion spots. Scale bars, 10 μm. **c,** Images for the case where the protein-rich phase is the continuous phase, and the protein-depleted phase is the dispersed phase (described as Case 3 in the main text). Scale bar, 2 μm. **d,e** Images showing coupled and uncoupled regions when protein access to both sides of the membrane was possible. Scale bars, 2 μm. **f-h,** Relative intensity profile along the dotted line in the merged channel in **d** for coupled (**f**) and uncoupled (**g**) regions, and in the merged channel in **e** (**h**), where green and red lines represent the intensity from protein and lipid channels, respectively. 1 μM of ENTH-RGG, labeled with Atto 488, was used. The same membrane composition (80 mol% DOPC, 15 mol% DOPS, 5 mol% PI(4,5)P2, and 0.5 mol% BODIPY TR Ceramide) and buffer (25 mM HEPES, 100 mM NaCl, pH 7.4) were used for all the panels.
