# Supplementary material for: Transmembrane coupling of liquid-like protein condensates
Source: Nat Commun. 2023 Dec 4;14:8015. doi: 10.1038/s41467-023-43332-w (PMC10696066; doi:10.1038/s41467-023-43332-w)
Supplement: Supplementary file 3 — Description of additional supplementary files [file 41467_2023_43332_MOESM3_ESM.pdf]

Description of additional supplementary files

Supplementary Movie 1. Protein phase separation on several hexagonal lipid membranes over time. 1 µM of his-RGG labeled with Atto 488 was added. The time on the upper left corner represents the elapsed time since protein addition. Membrane composition: 75 mol% DOPC, 25 mol% DGS-Ni-NTA, 0.5 mol% Texas Red-DHPE. Buffer: 25 mM HEPES, 100 mM NaCl, pH 7.4. Scale bar, 50 µm.

Supplementary Movie 2. Fusion events between protein-rich domains on the membrane over time. 1 µM of his-RGG labeled with Atto 488 was added. Membrane composition: 85 mol% DOPC, 15 mol% DGS-Ni-NTA, 0.5 mol% Texas Red-DHPE. Buffer: 25 mM HEPES, 100 mM NaCl, pH 7.4. Scale bar, 10 µm.

Supplementary Movie 3. Fusion events between protein-rich domains on the same side of the membrane over time, as shown in Figure 3d. 1 µM of his-RGG labeled with Atto 488 was added. Membrane composition: 75 mol% DOPC, 25 mol% DGS-Ni-NTA, 0.5 mol% Texas Red-DHPE. Buffer: 25 mM HEPES, 100 mM NaCl, pH 7.4. Scale bar, 5 µm.

Supplementary Movie 4. Transmembrane coupling of protein-rich domains on different sides of the membrane over time, as shown in Figure 3f. 1 µM of his-RGG labeled with Atto 488 was added. Membrane composition: 75 mol% DOPC, 25 mol% DGS-Ni-NTA, 0.5 mol% Texas Red-DHPE. Buffer: 25 mM HEPES, 100 mM NaCl, pH 7.4. Scale bar, 2 µm.

Supplementary Movie 5. Transmembrane coupling of protein-rich domains on different sides of the membrane over time, as shown in Figure 3i. 1 µM of his-RGG labeled with Atto 488 was added. Membrane composition: 75 mol% DOPC, 25 mol% DGS-Ni-NTA, 0.5 mol% Texas Red-DHPE. Buffer: 25 mM HEPES, 100 mM NaCl, pH 7.4. Scale bar, 2 µm.
